# Efficacy and safety of medical cannabinoids in children with cerebral palsy: a systematic review

**DOI:** 10.31744/einstein_journal/2023RW0387

**Published:** 2023-11-10

**Authors:** Widya Murni, Tungki Pratama Umar, Kevin Tandarto, Abraham Simatupang, Armedy Ronny Hasugian, Reza Yuridian Purwoko, Sri Idaiani, Bella Stevanny, Caroline Oktarina, Reganedgary Jonlean, Tamara Tango, Kevin Surya Kusuma, Sagita Pratiwi Sugiyono, Aditya Putra

**Affiliations:** 1 Jakarta Anti-Aging Center Clinic Jakarta Indonesia Jakarta Anti-Aging Center Clinic , Jakarta , Indonesia .; 2 Faculty of Medicine Sriwijaya University Palembang Indonesia Faculty of Medicine , Sriwijaya University , Palembang , Indonesia .; 3 Faculty of Medicine and Health Sciences Atma Jaya Catholic University of Indonesia Jakarta Indonesia Faculty of Medicine and Health Sciences , Atma Jaya Catholic University of Indonesia , Jakarta , Indonesia .; 4 Department of Pharmacology and Therapy Faculty of Medicine Universitas Kristen Indonesia Jakarta Indonesia Department of Pharmacology and Therapy , Faculty of Medicine , Universitas Kristen Indonesia , Jakarta , Indonesia .; 5 Indonesia National Research and Innovation Agency Jakarta Indonesia Indonesia National Research and Innovation Agency , Jakarta , Indonesia .; 6 Faculty of Medicine University of Indonesia Jakarta Indonesia Faculty of Medicine , University of Indonesia , Jakarta , Indonesia .

**Keywords:** Cannabinoids, Child, Cerebral palsy, Efficacy, Safety, Patient safety

## Abstract

**Introduction:**

The increasing popularity of cannabinoids for treating numerous neurological disorders has been reported in various countries. Although it reduces tetrahydrocannabinol psychoactivity, it helps patients tolerate higher doses and complements the anti-spasmodic effects of tetrahydrocannabinol. One of the most important potential of cannabinoids are related to its potential to help children with cerebral palsy, a contributor of lifelong disability. Therefore, this systematic review aimed to assess the efficacy and safety of medical cannabinoids in children with cerebral palsy.

**Methods:**

This review adhered to The Preferred Reporting Items for Systematic Reviews and Meta-analysis 2020 guidelines. Seven databases, namely, Scopus, PubMed, EBSCO Host, ProQuest, Google Scholar, Semantic Scholar, and JSTOR, were used to identify relevant studies. Studies examining pediatric patients with cerebral palsy and reporting the efficacy and safety of medical cannabinoids through clinical trials, observational cross-sectional studies, or cohort designs were included. The outcomes of the studies included the efficacy of medical cannabinoids administered for spasticity, motor components, pain control, sleep difficulties, adverse effects, and seizure control.

**Results:**

Of 803 identified articles, only three met the inclusion criteria for data synthesis. One study exhibited a moderate risk-of-bias. A total of 133 respondents, mainly from Europe, were investigated. Overall effectiveness and safety were considered good. However, the results are inconsistent, especially regarding spasticity treatment variables.

**Conclusion:**

The anti-spasticity, anti-inflammatory, and anti-seizure properties of cannabinoids might be beneficial for patients with cerebral palsy, although their effectiveness has not been widely studied. Further studies with larger sample sizes and various ethnicities are warranted. Prospero database registration: (www.crd.york.ac.uk/prospero) under ID CRD42022358383.

## INTRODUCTION

Complex movement disorders are a heterogeneous group of neurological disorders characterized by different types of abnormal movements and postures, including spasticity and dystonia. These abnormal movements and postures are generally associated with severe orthopedic problems, chronic pain, eating difficulties, constipation, sleep disturbances, epilepsy, and a poor quality of life. ^( 1 )^ Cerebral palsy (CP) is the most common complex movement disorder, with multiple complications that begin during childhood. The estimated prevalence is 2–3 per 1,000 live births. ^( 2 )^ Medical cannabinoids are becoming increasingly popular in several countries. Cannabinoid-based therapies have been investigated for the treatment of various neurological disorders, particularly drug-resistant epilepsy and movement disorders. ^( 3 )^ The methodologies used in these studies and the derived results are controversial. Cannabinoid-based drugs, phytocannabinoids, and synthetic cannabinoids have multiple mechanisms of action including interactions with endocannabinoid receptors. ^( 4 , 5 )^ In addition, cannabidiol may potentiate some of the beneficial effects of tetrahydrocannabinol (THC) as it reduces the psychoactivity of THC and allows patients to tolerate higher THC doses. Cannabidiol may also complement the anti-spasmodic effects of THC ( *e.g* ., via local enhancement of glycine signaling; inhibition of endocannabinoid degradation; or delayed demyelination through anti-inflammatory, antioxidant, and anti-excitotoxic mechanisms). ^( 5 )^ Cannabinoids have therapeutic potential in movement disorders. Synthetic cannabinoids, such as nabilone, dronabinol, and Sativex, are cannabinoid receptor agonists with effects similar to those of THC. They have been approved for clinical indications including spasticity, pain, and refractory epilepsy. ^( 6 , 7 )^ However, its efficacy and safety in children with CP are uncertain, especially in the treatment of spasticity, pain, and seizures. This systematic review aimed to assess the efficacy and safety of medical cannabinoids in children with CP.

## METHODS

### Study registration and strategy

The Preferred Reporting Items for Systematic Reviews and Meta-Analysis 2020 guidelines were followed to develop this review. The Scopus, PubMed, EBSCO Host, ProQuest, Google Scholar, Semantic Scholar, and JSTOR databases were used to identify studies relevant to the search terms. The search terms used in each database are listed in Appendix 1 . No time restrictions were imposed on the literature search. Manual search was also conducted by examining the citations of selected articles to identify relevant publications that were not indexed in the aforementioned databases. ^( 8 )^

### Eligibility criteria

Studies examining pediatric patients with CP and reporting the efficacy and safety of medical cannabinoids through clinical trials, observational cross-sectional studies, or cohort designs were included. Systematic reviews, meta-analyses, narrative reviews, case reports, case series, opinion pieces, conference abstracts, and grey literature were excluded.

### Study selection

The titles and abstracts of unique studies were independently screened using Rayyan QCRI, an online software used for abstract and title screening. ^( 9 , 10 )^ This process was performed by two reviewers, and supervised and adjudicated, if necessary, by a single reviewer. Full-text articles were obtained and two reviewers independently conducted eligibility assessments. No reviewers were blinded to the bibliographic information of the studies.

### Outcomes

The outcomes of the studies included the efficacy of medical cannabinoids administered for spasticity, motor components, pain control, sleep difficulties, adverse effects, and seizure control. Cerebral palsy should be assessed using validated tests and internationally standardized diagnostic criteria.

### Risk-of-bias

Several tools were used to assess the risk-of-bias based on the study type. We used the Risk-of-Bias in Non-Randomized Intervention Studies tool (ROBINS-I) to examine the potential risk-of-bias in the selected experimental nonrandomized studies. The tool can be assigned for the following areas: (i) entanglement bias, (ii) study participant selection bias, (iii) exposure measurement bias, (iv) exposure misclassification bias during follow-up, (v) bias in outcome measurement, (vi) bias in missing data, and (vii) bias in the selection of the results. The risk-of-bias was assessed on 0–4 scale points following the severity of the bias risk. The randomized studies used the RoB-2 tool. The five following domains were assessed: i) randomization, ii) deviation from the intended intervention, iii) outcome measures, iv) missing outcome data, and v) selection from reported outcomes. Furthermore, we assessed the potential bias in cross-sectional studies using the Risk-of-Bias Instrument for Cross-sectional Surveys of Attitudes and Practices contributed by the CLARITY group at McMaster University. The following five domains were assessed: i) representative population, ii) adequate response rate, iii) missing data, iv) clinically sound, and v) reliability and validity of the survey instrument.

Two reviewers independently assessed the risk-of-bias and the strength of evidence in all relevant studies. Any disagreements between the two researchers were resolved through consultation with a third researcher.

### Ethics approval

The authors of this article did not conduct human or animal studies. Therefore, ethical approval was not required.

### Patient and publication consent statement

The authors of this article did not conduct human or animal studies. Therefore, patient and publication consent was not required.

## RESULTS

### Study screening

**Figure 1:**
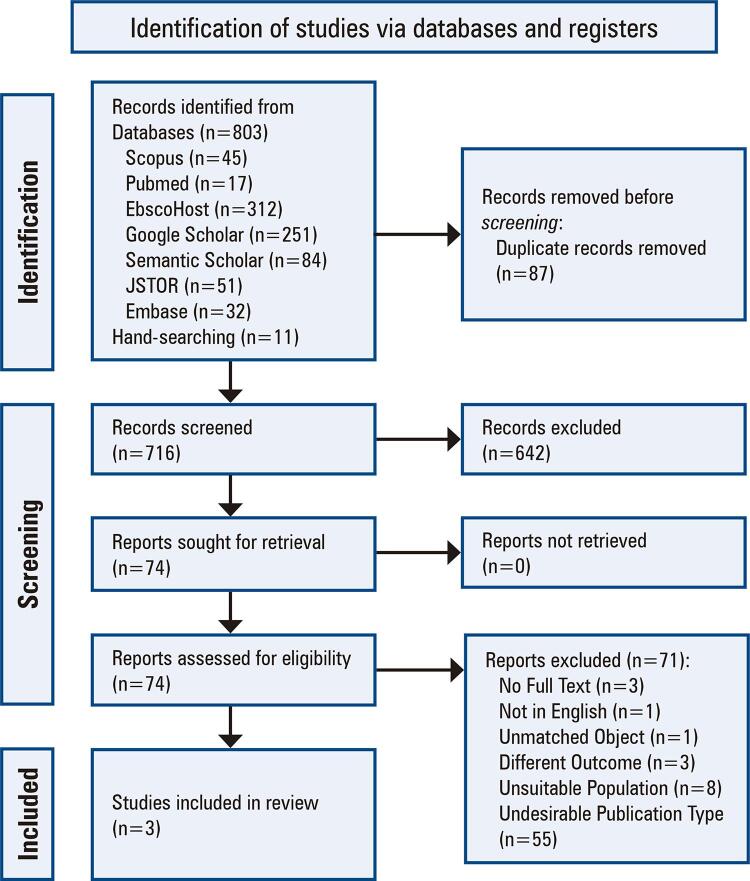
Study flow diagram

The search strategy yielded 803 articles, of which the titles and abstracts of 716 unique articles were evaluated for eligibility after deduplication. A total of 642 articles were considered ineligible and were excluded in the first screening phase. The full texts of the remaining 74 articles were reviewed to assess their eligibility for inclusion and 71 articles were excluded. Three studies met the inclusion criteria for data synthesis ( Figure 1 ).

### Study characteristics

Table 1 presents the characteristics of the included studies. This systematic review included three research projects, one of which was an observational study and two were experimental studies (RCT and non-RCT). There were 133 respondents (range: 19–70 participants). Each study included a European population. ^11^ All investigations examined the benefits and adverse effects of medical cannabis administration. Only two studies provided information on the average age of participants and duration of treatment, with a mean ages of 12.6 ^( 11 )^ and 6.51 ^( 12 )^ years and treatment durations of 12 weeks ^( 11 )^ and five months, ^( 12 )^ respectively.

**Table 1:** Summary of included studies

Author	Country	Study type	Sample size	Comparison	Mean Age (years)	Duration of treatment	Substance	Instrument	Main findings
Fairhurst et.al. ^( 11 )^ (2020)	United Kingdom, Israel, Czech Republic	RCT	72 (CP: 47)	Placebo control	12.6	12 weeks	Oromucosal nabiximols	NRS for spasticity	No significant difference in the spasticity between nabiximols versus placebo groups after 12 weeks (p=0.729) The substance is generally well tolerated by pediatric patients, with three cases of hallucinations
Libzon et.al. ^( 12 )^ (2018)	Israel	Non-RCT	25 (CP: 19)	No Control Group	6.51	5 months	Cannabidiol-enriched 5% oil formulation (cannabidiol-to-THC ratio 6:1 and 20:1)	NRS for spasticity, VAS for pain, dystonia scale, CPCHILDQoL questionnaire	NRS for spasticity improved from baseline in the entire study population regardless of treatment assignment. VAS score improved in addition to pain duration, frequency, and dystonia. The CPCHILDQoL improved in the study cohort. Adverse effects were rarely reported (including seizure deterioration). There were no changes in ECG or blood tests
Morosoli et.al. ^( 13 )^ (2021)	Europe, North America, Australia	Cross-sectional	70	N/A	N/A	N/A	Medical cannabinoids (including cannabis oil, dronabinol solution, cannabis tincture, and cannabis spray)	Questionnaire	The impact of medical cannabinoids on CP symptoms alleviation is mainly considered strong or moderate (68%) Common acute side effects of cannabinoid administration are sleepiness, restlessness, diarrhea, and nausea. No long-term side effects are witnessed

CP: cerebral palsy; CPCHILDQoL: Cerebral Palsy Child Questionnaire for Quality of Life; ECG: electrocardiogram; NRS: numeric rating scale; RCT: Randomized Controlled Trial; VAS:visual analog scale.

### Types of medical cannabinoids and administration methods

All studies used various cannabinoid substances and administration methods. Fairhurst et al. ^( 11 )^ used a nabiximols solution containing 2.7mg THC, 2.5mg cannabidiol (CBD), and other cannabinoid and non-cannabinoid components. The medications were administered orally or sublingually for 12 weeks. Meanwhile, Libzon et al. ^( 12 )^ used two products of CBD nourished with 5% oil preparation (one with CBD: THC=20:1 and the other with CBD: THC=6:1), Which were administered twice or thrice daily for five months via an oral or feeding tube route. Furthermore, an observational study by Morosoli et al. ^( 13 )^ revealed that only 52% of patients specified a particular cannabinoid prescription, which included dronabinol solution, cannabis oil, cannabis, *Cannabis sativa* spray, CBD, and Epidiolex.

### Role of medical cannabis in cerebral palsy symptoms alleviation

The effects of medical cannabis on patient complaints varied. Fairhurst et al. ^( 11 )^ found no difference in the primary outcome of caregiver-reported spasticity between the cannabis-administered and control groups (mean difference: -0.166, p=0.729). In contrast, Libzon et al. ^( 12 )^ showed that irrespective of treatment allocation, the entire study population showed improved spasticity, quality of life (QOL), and gross motor coordination. Along with pain duration and frequency, pain intensity decreased, as evidenced by an improvement in the visual analog scale (VAS) score. Meanwhile, Morosoli et al. ^( 13 )^ reported that, while not specifically addressing the domain of improvement, more than half of the participants experienced a moderate-to-strong influence from cannabis administration, with about one-fifth reporting no improvement.

### Safety profile of medical cannabis in cerebral palsy patients

Medical cannabis is particularly safe for pediatric patients with CP. The adverse events were mild-to-moderate, with no long-term consequences. ^( 13 )^ Tiredness, dizziness, exhaustion, xerostomia, diarrhea, nausea, vomiting, and confusion are several examples of reported symptoms. Serious adverse events included changes in seizure characteristics, ^( 11 , 12 )^ disorientation, euphoria, hypotonia, distress, hallucinations, psychotic symptoms, food aversion, elevated liver enzymes, and viral upper respiratory tract infections. ^( 11 , 13 )^ In contrast, Libzon et al. did not report any deterioration in the electrocardiogram (ECG) or blood tests, and the detrimental reactions could be controlled by reducing the dose of cannabinoids. ^( 12 )^

### Study quality (risk-of-bias)

**Figure 2:**
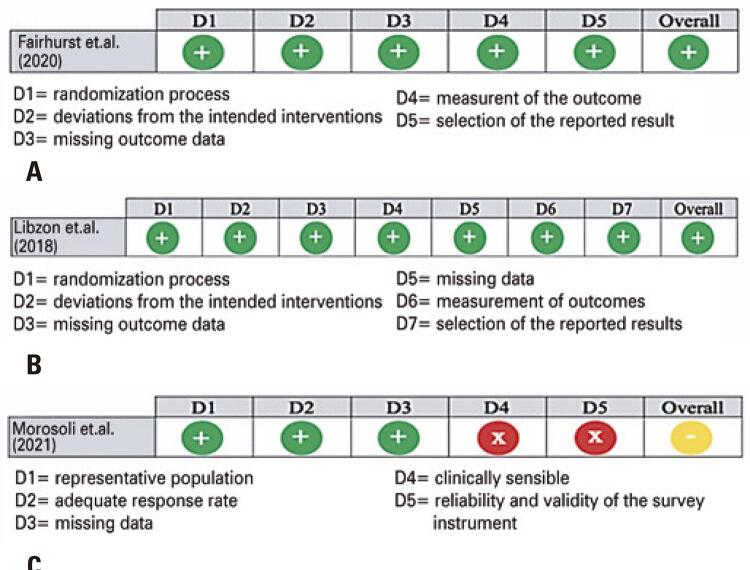
Risk of Bias Assessment. (A) Cochrane risk-of-bias tool for randomized trials risk-of-bias 2; (B) Risk of bias in non-randomized studies - of interventions (ROBINS-I); (C) Risk of Bias for Cross-Sectional Surveys of Attitudes and Practices

The overall risk-of-bias was moderate-to-low. The moderate results in the study by Morosoli et al. ^( 13 )^ were related to the lack of adequate clinical assessment and the reliability and validity of the survey instrument domains. This was not observed in the other included studies. The risk-of-bias assessment is shown in figure 2 .

## DISCUSSION

Reports on the benefits of medical cannabis are increasing. Despite the controversy over its safety and efficacy, studies have suggested that cannabis may have the therapeutic potential to improve several disorders, including neurological diseases. ^14^ Reduction in seizure frequency, ^( 17 , 18 )^ spasticity, neuropathic pain, ^( 15 )^ and other motor function disorders ^( 16 , 18 )^ are the most-reported positive impacts.

However, concerns regarding the adverse effects of cannabis have emerged. Non-serious adverse effects may occur even before cannabis initiation. ^11^ However, they can be generally resolved by reducing the dose of cannabis or changing the pattern of administration. ^( 12 , 16 , 17 )^ THC is the most important contributor to the psychoactive side-effects. Using a ‘start low and go slow’ dosing strategy and combining CBD with THC may mitigate most adverse events. ^( 19 )^ Therefore, the safety level is generally acceptable if the medication dose and administration are suitable.

Cannabis plants contain more than 100 known cannabinoid compounds. The psychoactive Δ9-tetrahydrocannabinol (Δ9-THC) and the non-psychoactive CBD are the principal neuroactive components of cannabis. The term “non-psychoactive” refers to the absence of psychotropic effects compared to Δ9-THC. ^( 20 )^ Most cannabis retail products fall into one of the following three categories: Δ9-THC-dominant, CBD-dominant, or a balanced “hybrid” product with high concentrations of both Δ9-THC and CBD. ^( 21 )^ These products can be consumed through several methods, including smoke inhalation, vaporization, oral ingestion, and other routes (topical and suppository). ^( 19 )^ All three included studies reported on oral-ingestion of various preparations of cannabis, such as oils, tinctures, and sprays, which are associated with better convenience and less odor. ^( 22 )^

Cerebral palsy refers to a set of persistent movements and postural disorders that restrict activity and is caused by non-progressive disruptions in the developing brain. ^( 23 )^ This disorder causes motor disorders, a broad range of comorbidities, and secondary conditions such as sensation, perception, cognition, communication, and behavioral disturbances. ^( 24 )^ Pain is the most common secondary consequence of severe health concerns in children with CP. It is primarily related to movement disorders (the basis of CP), musculoskeletal problems, and repeated exposure to painful procedures, including surgery. ^( 25 )^

The effect of cannabinoid administration in patients with CP is uncertain despite its high potential. ^( 26 )^ There are two types of cannabinoid receptors in human cells: types 1 (CB1) and 2 (CB2). CB1 is found in the central nervous system and peripheral tissues, whereas CB2 is mainly found in the immune cells. ^( 27 )^ CB1 receptors are primarily located at the terminals of the central and peripheral neurons, where they regulate neurotransmitter release and psychoactive traits. They are also abundant in brain areas associated with nociceptive perception, such as the thalamus and amygdala. ^( 28 )^ CB1 receptor activation modifies nociceptive thresholds and exerts various biological effects by balancing excitatory and inhibitory neurotransmitters, mainly related to the Gamma-Aminobutyric Acid system, which can be used to treat seizures and spasms. ^( 29 , 30 )^ CB2 receptor activation hinders the release of inflammatory mediators by cells close to the nociceptive nerve terminals and prevents pain signals from entering the central nervous system. ^( 28 )^

Cannabinoids presynaptically hinder glutamate release. ^( 31 )^ The amplification of CB1 receptors reduces glutamatergic transmission in THC-exposed animals, and chronic cannabis use decreases glutamate metabolites in the human brain. Several animal studies have identified CB1 as a receptor that modulates the anti-spasticity effects of cannabinoids. ^( 28 )^ Owing to its effectiveness in alleviating spasticity in both animal models and humans, Δ9-THC is the most important cannabinoid that mediates the anti-spasticity effect of cannabinoid preparations. ^( 32 )^ Therefore, Δ9-THC is responsible for treating CP-related dystonia.

**Figure 3:**
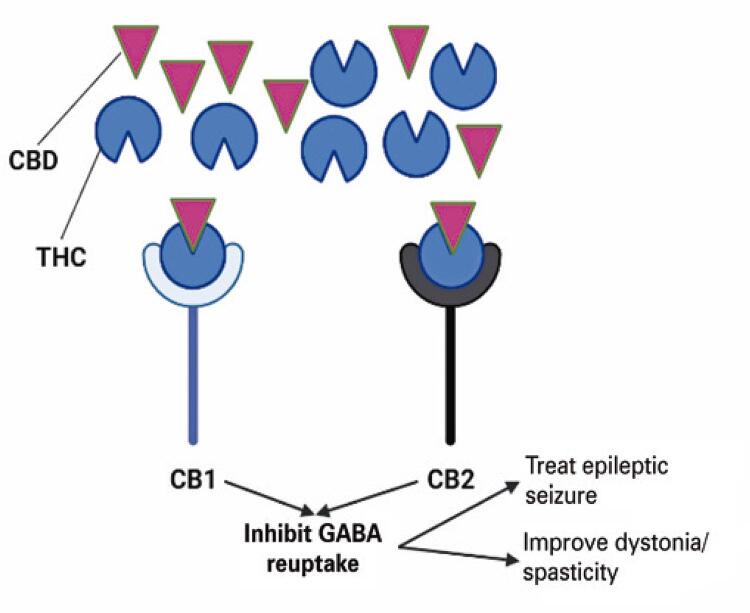
Mechanism of cannabidiol and tetrahydrocannabinol in Improving cerebral palsy-symptoms

CBD: cannabidiol; CB1: cannabinoid receptor type 1; CB2: cannabinoid receptor type 2; GABA: gamma-aminobutyric acid; THC, tetrahydrocannabinol.

In addition to lowering spasticity, Δ9-THC and CBD have been shown to prevent seizures in animal models. These substances exhibit low toxicity and high tolerability. ^( 33 )^ Δ9-THC is a partial agonist of both CB1 and CB2 receptors and reduces the severity of seizures by activating CB1. Furthermore, Δ9-THC has powerful anti-inflammatory effects on microglia, which are the primary immune cells of the central nervous system. Considering the synergistic relationship between seizures and inflammation, the cannabinoid system offers a novel strategy for targeting both sectors of this feedback mechanism. ^( 34 )^ Cannabidiol structurally resembles Δ9-THC, but has low affinity for CB1 and CB2 receptors. Cannabidiol may exert anti-seizure effects by reducing glutamate release. CBD also minimizes epileptiform events in the hippocampus in an in vitro model in a CB1-independent, concentration-dependent, and region-specific manner. ^( 35 )^ The illustration of the effects of cannabinoids on spasticity and epileptic seizures is shown in figure 3 .

## LIMITATIONS

This systematic review has some limitations. The number of studies included in this systematic review was limited to those with different study designs. Of the three studies, only one was an RCT. The small sample sizes of the included studies may not represent real-world efficacy and safety. In addition, the study focused mainly on the European population, making generalizability challenging to achieve.

## CONCLUSION

Cannabinoids may be beneficial in patients with cerebral palsy; however, their effectiveness has yet to be thoroughly studied. The proposed modes of action of cannabinoids in cerebral palsy include anti-spasticity, anti-inflammatory, and anti-seizure features. Although mild-to-moderate adverse events have been reported, there have been no reports of long-term adverse events, indicating a favorable safety profile. Further research with a larger sample size, extended study period, and individuals of various ethnicities is required to determine the role of cannabinoids in cerebral palsy.
