# Supplementary material for: Efficacy and safety of medical cannabinoids in children with cerebral palsy: a systematic review
Source: Einstein (Sao Paulo). 2023 Nov 10;21:eRW0387. doi: 10.31744/einstein_journal/2023RW0387 (PMC10691312; doi:10.31744/einstein_journal/2023RW0387)
Supplement: Supplementary file 1 [file 2317-6385-eins-21-eRW0387-suppl01.pdf]

Appendix 1. Search terms for each database

Database	Search term
Scopus	("Cerebral palsy" OR "spastic paralysis" OR "Static Encephalopathy" OR "congenital diplegia" OR "spastic diplegia" OR "congenital quadriplegia" OR "spastic quadriplegia" OR "congenital hemiplegia" OR "spastic hemiplegia" OR " Choreoathetoid Cerebral Palsy" OR "extrapyramidal cerebral palsy" OR "dyskinetic cerebral palsy") AND ("cannabis" OR "medical cannabis" OR "cannabinoids" OR "medical cannabinoids" OR "marijuana" OR "hemp" OR "hashish" OR "Tetrahydrocannabinol" OR "cannabinol") AND ("effectiveness" OR "outcome" OR "benefit" OR "spasticity" OR "dystonia" OR "sleep difficulties" OR "sleep problems" OR "pain" OR "seizure" OR "epilepsy" OR "safety" OR "mortality" OR "adverse effects" OR "adverse events" OR "behavioral changes" OR "psychiatric problems")
Pubmed	("Cerebral palsy" OR "spastic paralysis" OR "Static Encephalopathy" OR "congenital diplegia" OR "spastic diplegia" OR "congenital quadriplegia" OR "spastic quadriplegia" OR "congenital hemiplegia" OR "spastic hemiplegia" OR " Choreoathetoid Cerebral Palsy" OR "extrapyramidal cerebral palsy" OR "dyskinetic cerebral palsy") AND ("cannabis" OR "medical cannabis" OR "cannabinoids" OR "medical cannabinoids" OR "marijuana" OR "hemp" OR "hashish" OR "Tetrahydrocannabinol" OR "cannabinol") AND ("effectiveness" OR "outcome" OR "benefit" OR "spasticity" OR "dystonia" OR "sleep difficulties" OR "sleep problems" OR "pain" OR "seizure" OR "epilepsy" OR "safety" OR "mortality" OR "adverse effects" OR "adverse events" OR "behavioral changes" OR "psychiatric problems")
Proquest	("Cerebral palsy" OR "spastic paralysis" OR "Static Encephalopathy") AND ("cannabis" OR "medical cannabis" OR "cannabinoids" OR "medical cannabinoids") AND ("effectiveness" OR "outcome" OR "adverse effects" OR "adverse events")
Ebsco	("Cerebral palsy" OR "spastic paralysis" OR "Static Encephalopathy") AND ("cannabis" OR "medical cannabis" OR "cannabinoids" OR "medical cannabinoids") AND ("effectiveness" OR "outcome" OR "adverse effects" OR "adverse events")
Google Scholar	"Cerebral palsy", "cannabis", "cannabinoids", "effectiveness", "outcome", "safety"
JSTOR	("Cerebral palsy" OR "spastic paralysis" OR "Static Encephalopathy") AND ("cannabis" OR "medical cannabis" OR "cannabinoids" OR "medical cannabinoids") AND ("effectiveness" OR "adverse effects")
Semantic Scholar	"Cerebral palsy", "cannabis", "cannabinoids", "effectiveness", "efficacy", "safety"
